# Identification of HLA-A2 or HLA-A24-restricted CTL epitopes for potential HSP105-targeted immunotherapy in colorectal cancer

**DOI:** 10.3892/or.2013.2941

**Published:** 2013-12-20

**Authors:** YU SAWADA, HIROYUKI KOMORI, YOSHIYUKI TSUNODA, MANAMI SHIMOMURA, MARI TAKAHASHI, HIDEO BABA, MASAAKI ITO, NORIO SAITO, HIROYUKI KUWANO, ITARU ENDO, YASUHARU NISHIMURA, TETSUYA NAKATSURA

**Affiliations:** 1 Division of Cancer Immunotherapy, Exploratory Oncology Research and Clinical Trial Center, National Cancer Center, Kashiwa 277-8577, Japan; 2 Department of Gastroenterological Surgery, Yokohama City University Graduate School of Medicine, Yokohama 236-0004, Japan; 3 Department of Immunogenetics, Graduate School of Medical Sciences, Kumamoto University, Kumamoto 860-8556, Japan; 4 Department of Gastroenterological Surgery, Graduate School of Medical Sciences, Kumamoto University, Kumamoto 860-8556, Japan; 5 Colorectal and Pelvic Surgery Division, National Cancer Center Hospital East, Kashiwa 277-8577, Japan; 6 Department of General Surgical Science (Surgery I), Gunma University Graduate School of Medicine, Maebashi 371-8511, Japan

**Keywords:** cancer immunotherapy, cytotoxic T cell, colorectal cancer, heat shock protein 105, HLA-transgenic mice

## Abstract

We previously reported that heat shock protein 105 (HSP105) is overexpressed in a variety of human cancers, including colorectal, pancreatic and esophageal cancer and has proven to be a novel biomarker for the immunohistochemical detection of these cancers. In the present study, we used HLA-transgenic mice (Tgm) and the peripheral blood mononuclear cells (PBMCs) of colorectal cancer patients to identify HLA-A2 and HLA-A24-restricted HSP105 epitopes, as a means of expanding the application of HSP105-based immunotherapy to HLA-A2- or HLA-A24-positive cancer patients. In addition, we investigated by *ex vivo* IFN-γ ELISPOT assay whether the HSP105-derived peptide of cytotoxic T cells (CTLs) exists in PBMCs of pre-surgical colorectal cancer patients. We found that four peptides, HSP105 A2-7 (RLMNDMTAV), HSP105 A2-12 (KLMSSNSTDL), HSP105 A24-1 (NYGIYKQDL) and HSP105 A24-7 (EYVYEFRDKL), are potential HLA-A2 or HLA-A24-restricted CTL HSP105-derived epitopes. HSP105-specific IFN-γ-secreting T cells were detected in 14 of 21 pre-surgical patients with colorectal cancer in response to stimulation with these four peptides. Our study raises the possibility that these HSP105 peptides are applicable to cancer immunotherapy in patients with HSP105-expressing cancer, particularly colorectal cancer.

## Introduction

Colorectal cancer is one of the most prevalent cancers and a major cause of mortality worldwide (1). Although adjuvant systemic chemotherapy or chemoradiation can confer a limited but significant survival advantage, novel and more effective therapies are needed. To improve survival rates, new therapeutic agents have been investigated. Immunotherapy for colorectal cancer is a promising candidate treatment, and there is evidence that host immune responses can influence survival (2). Ideal targets for immunotherapy are gene products overexpressed in cancer cells but silenced in normal tissues, with the exception of immune-privileged tissues, such as that of the testis.

We previously reported that heat shock protein 105 (HSP105), identified by SEREX, is overexpressed in a variety of human cancers, including colorectal, pancreatic and esophageal cancer, but with little to no expression in normal tissues aside from the testis (3,4). HSP105 is a stress protein induced by various stressors and belongs to the HSP105/110 family and plays an important role as a chaperone under physiological conditions (5). Using immunohistochemical analysis, we previously found that HSP105 was specifically overexpressed in 44 of 53 (83.0%) colorectal cancer patients (4). It has also been reported that DNA vaccination with both HSP105 and bone marrow-derived dendritic cells (BM-DCs) pulsed with HSP105 led to tumor rejection of colorectal cancer but did not induce an autoimmune reaction in mice (6–8).

This suggests that HSP105 presents a useful tumor-specific antigen target for immunotherapy. However, HSP105-derived epitope peptides of CD8^+^ T cells have not been identified. The gene frequency of HLA-A24 (A*24:02) is relatively high in Asian populations, especially the Japanese, but low in Caucasians. On the other hand, the gene frequency of HLA-A2 (A*02:01) is high among several ethnic groups, including Asians and Caucasians (9). Therefore, HLA-A2 or HLA-A24-restricted cytotoxic T cell (CTL) HSP105 epitopes could be extremely useful for immunotherapy in a large portion of patients worldwide. In the present study, we identified human HSP105-derived CTL epitopes restricted by HLA-A2 or HLA-A24 using HLA-transgenic mice (Tgm) and examined whether these epitope-based peptides could activate HSP105-reactive CTLs in peripheral blood mononuclear cells (PBMCs) of patients with colorectal cancer.

## Materials and methods

### Mice

HLA-A2.1 (HHD) Tgm, H-2D^b−/−^β2m^−/−^ double-knockout mice introduced with the human β2m-HLA-A2.1(α1 α2)-H-2D^b^ (α3 transmembrane cytoplasmic) (HHD) mono-chain gene construct were generated in the Departmente SIDA-Retrovirus, Unite d’ Immunite Cellulaire Antivirale, Institut Pasteur, Paris, France (10,11) and were kindly provided by Dr F.A. Lemonier. HLA-A24.2 (HHD) Tgm were purchased from Japan SLC, Inc. (Shizuoka, Japan). Female 6- to 8-week-old BALB/c mice (H-2K^d^) and BALB/c nude mice, purchased from Charles River Japan (Yokohama, Japan), were maintained and handled in accordance with animal care policy.

### Cell lines

The human colorectal cancer cell line SW620 (endogenously expressing HSP105 and HLA-A*02:01, 24:02) and human liver cancer cell line HepG2 (HSP105-low expressing and HLA-A*02:01, 24:02), were kindly provided by the Cell Resource Center for Biomedical Research, Institute of Development, Aging and Cancer (Tohoku University, Sendai, Japan). Murine colorectal cancer cells, Colon26 (C26) (endogenously expressing HSP105 and H-2K^d^) were kindly provided by Dr Kyoichi Shimomura (Fujisawa Pharmaceutical Co., Osaka, Japan). T2 cells (a TAP-deficient and HLA-A*02:01-positive cell line) were provided by Kyogo Ito of Kurume University. Cells were maintained *in vitro* in RPMI-1640 or DMEM supplemented with 10% FCS.

### RNA interference

Small interfering RNAs targeting human HSP105 were chemically synthesized by Dharmacon Research (HSP105-siRNA and luciferase; Lafayette, CO, USA) as previously described (12), with the following siRNA sequences: HSP105-siRNA, UUGGCUGCAACUCCGAUU GTT and luciferase, CGUACGCGGAAUACUUCGATT. The transfection of siRNA oligonucleotides was carried out using Oligofectamine (Invitrogen, Carlsbad, CA, USA) according to the manufacturer’s guidelines.

### Peptides

Human HSP105-derived peptides, identical in amino acid sequence with mouse HSP105 and expressing the binding motifs for HLA-A*02:01- and HLA-A*24:02-encoded molecules, were designed with BIMAS software (BioInformatics and Molecular Analysis Section; Center for Information Technology, NIH, MD, USA). We purchased a total of 16 versions of peptides carrying the HLA-A2 (A*0201)-binding motifs and 9 versions of peptides carrying the HLA-A24 (A*2402)-binding motifs from Biologica (Tokyo, Japan) (Table I).

### Induction of HSP105-reactive CTLs in Tgm

Peptide immunizations in mice were performed as previously described (13). In brief, bone marrow (BM) cells (2×10^6^) from HLA-A2 or HLA-A24 Tgm were cultured in RPMI-1640 medium supplemented with 10% FCS, GM-CSF (5 ng/ml) and 2-mercaptoethanol (0.8 ng/ml) for 7 days in 10-cm plastic dishes. These BM-DCs were pulsed with the two HSP105 peptide mixtures (1 μmol/l each peptide) for 2 h at 37°C. We primed the HLA-A2 or HLA-A24 Tgm with the syngeneic BM-DC vaccine (5×10^5^/mice) into the peritoneal cavity twice, once per week. Seven days following the last immunization, the spleens were collected and CD4^−^ spleen cells were isolated by negative selection with anti-CD4 microbeads (Miltenyi Biotec, Bergisch Gladbach, Germany) to exclude any nonspecific IFN-γ production from the CD4^+^ spleen cells co-cultured with the BM-DCs. The CD4^−^ spleen cells (2×10^6^/well) were stimulated with syngeneic BM-DCs (2×10^5^/well) that had been pulsed with each peptide *in vitro*. After 6 days, the frequency of cells producing IFN-γ/2×10^4^ CD4^−^ spleen cells upon stimulation with syngeneic BM-DCs (1×10^4^/well), pulsed with or without each peptide, was assayed using an enzyme-linked immunospot (ELISPOT) assay as previously described (13).

### Identification of a CTL epitope in BALB/c mice

The peptide immunizations in mice were performed as previously described (14). Splenocytes removed from mice 7 days following the last immunization were harvested and cultured in 24-well culture plates (2.5×10^6^/well) in 45% RPMI, 45% AIMV, 10% FCS and supplemented with recombinant human interleukin 2 (100 U/ml), 2-mercaptoethanol (50 μmol/l) and each peptide (10 μmol/l). After 5 days, the cytotoxicity of these cells against target cells was assayed using standard 6-h ^51^Cr release assays (15).

### Blood samples

Blood samples from cancer patients were collected during routine diagnostic procedures after obtaining formal consent from patients at the Kumamoto University Hospital, from April to September 2006 and from patients at the National Cancer Center Hospital East, from December 2006 to March 2007. The study was approved by the local ethics committee, and informed consent was obtained from all patients.

### Induction of HSP105-reactive human CTLs

We isolated PBMCs from heparinized blood of HLA-A24^+^ and/or HLA-A2^+^ Japanese patients with colorectal cancer using Ficoll-Conray density gradient centrifugation; peripheral monocyte-derived dendritic cells (DCs) were generated as previously described (16,17). CD8^+^ T cells were isolated with CD8 microbeads (Miltenyl Biotec, Bergisch Gladbach, Germany) from PBMCs of the same donor and peptide-reactive CD8^+^ CTLs were generated. Five days following the last stimulation, the cytotoxic activities of the CTLs against cancer cell lines were measured by ^51^Cr-release assay as previously described (15). For these assays, CTLs were co-cultured with each cancer cell line, as the target cells (5×10^3^/well), at the indicated effector/target ratio.

### In vivo tumor challenge

Subcutaneous tumors were induced in mice by injecting 1×10^4^ SW620 cells suspended in 100 μl PBS or Hanks’ balanced salt solution (Gibco, Grand Island, NY, USA) into the backs of BALB/c nude mice. Tumor incidence and volumes were assessed weekly using calipers and tumor areas were measured. Results are presented as mean tumor areas ± SD.

### Ex vivo IFN-γ ELISPOT assay in peripheral blood in pre-surgical colorectal cancer patients

*Ex vivo* IFN-γ ELISPOT assays were performed to determine tumor-specific interferon-γ (IFN-γ)-secreting T cells. The 96-well plates were coated with anti-human IFN-γ (BD Biosciences Co., Ltd., USA). After an overnight incubation at 4°C, the wells were washed and blocked with complete medium for 2 h at room temperature. A total of 1×10^6^ unfractionated PBMCs were added in duplicate wells and incubated at 37°C for 18–20 h with or without peptides at 0.2 μl/well (1–10 μM). The plate was washed and then incubated with 5 μg/ml biotinylated anti-human IFN-γ antibody for 2 h at room temperature. After washing away the antibodies, streptavidin-HRP was added for 1 h. Finally, the plate was washed and replaced with fresh substrate solution and the reaction was terminated by washing with distilled water. The HLA-A2-restricted CMV peptide (NLVPMVATV) and HLA-A24 restricted CMV peptide (QYDPVAALF), which includes an epitope derived from the CMV pp65 protein, were used as positive controls.

### Histological and immunohistochemical analysis

To investigate whether CD8^+^ T cells infiltrated normal tissues triggered by the HSP105-derived peptide vaccine, we performed immunohistochemical staining with a monoclonal antibody against CD8 (1:100; LifeSpan BioSciences, Inc., Seattle, WA, USA) in tissue specimens from HLA-A2 Tgm immunized with the HSP105 peptides, as previously described (7). Immunohistochemical staining with rabbit polyclonal antibodies against HSP105 (1:200; Santa Cruz Biotechnology, Inc.; Santa Cruz, CA, USA) was performed according to the manufacturer’s instructions.

## Results

### Identification of HLA-A2-or HLA-A24-restricted CTL epitopes in HLA Tgm

**Figure 1:**
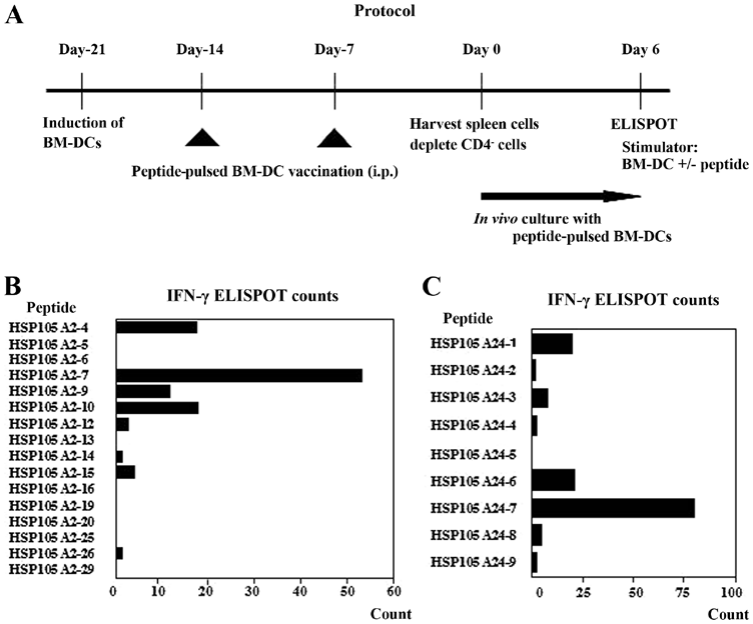
Identification of HLA-A2 or HLA-A24-restricted CTL epitopes of HSP105 using HLA-A2.1 Tgm and HLA-A24 Tgm. (A) The protocol used for identification of HLA-A2 or HLA-A24-restricted CTL epitopes of HSP105 is shown. We primed the HLA Tgm with BM-DCs (5×10^5^) pulsed with the mixture of HSP105-derived peptides carrying the HLA-A2 or HLA-A24 binding motif into the peritoneal cavity once a week for 2 weeks. Seven days after the last DC vaccination, spleens were collected and CD4^−^ spleen cells (2×10^6^/well) were stimulated with syngeneic BM-DCs (2×10^5^/well) pulsed with each peptide *in vitro* for 6 days. We used these cultured CD4^−^ spleen cells as responder cells in the IFN-γ ELISPOT assay. (B) The bar graphs show the IFN-γ ELISPOT counts per 2×10^4^ CD4^−^ spleen cells co-cultured with HLA-A2-restricted peptide-pulsed BM-DCs after normalization to counts from cells co-cultured with BM-DCs without peptide loading. (C) The bar graphs show the IFN-γ ELISPOT counts in the HLA-A24-restricted peptides. The columns represent the means from duplicate assays.

We designed pools of HSP105 peptides possessing amino acid sequences conserved between humans and mice that have a highly predicted binding score to HLA-A2 (pool of 16 different peptides) or HLA-A24 (A*24:02) (pool of 9 different peptides) (Table I). CD4^−^ spleen cells were obtained from Tgm immunized twice i.p. with BM-DCs that had been pulsed with each peptide mixture; the spleen cells were then stimulated *in vitro*, again with the BM-DCs pulsed with each peptide mixture (Fig. 1A).

The IFN-γ ELISPOT counts, normalized to those of spleen cells co-cultured with BM-DCs without peptide loading, clearly indicated a HSP105 A2-7 peptide-specific response in the CD4^−^ spleen cells (Fig. 1B). These CD4^−^ spleen cells (2×10^4^/well) showed 55±29.7 spot counts/well in response to the BM-DCs pulsed with the HSP105 A2-7 peptide, whereas they showed 23±31.1 spot counts/well in the presence of BM-DCs pulsed with the HSP105 A2-4 peptide. A similarly strong response was observed for the HSP105 A24-7 peptide (Fig. 1C). CD4^−^ spleen cells (2×10^4^/well) showed 79.5±27.6 spot counts/well in response to the BM-DCs pulsed with the HSP105 A24-7 peptide, whereas they showed 20.5±14.8 spot counts/well in the presence of BM-DCs with the HSP A24-6 peptide. These assays were performed twice with similar results and they suggest that the HSP105 A2-7 and A24-7 peptides are potential CTL epitope peptides in both HLA Tgm and humans.

### Identification of a CTL epitope in BALB/c mice and CTLs that are cytotoxic against C26 tumors in mice

**Figure 2:**
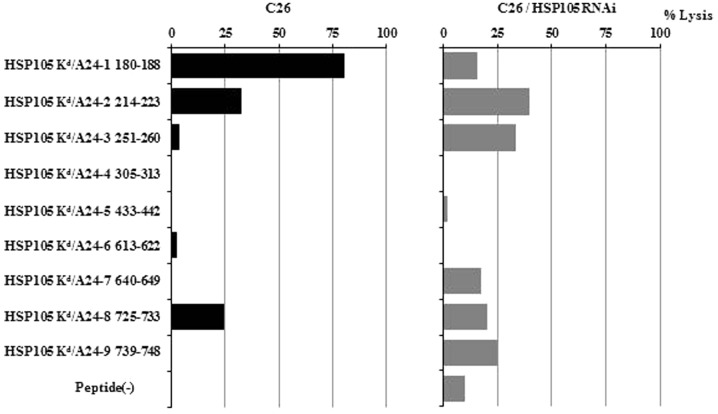
Identification of an HSP105-derived HLA-A24 and K^d^-restricted CTL epitope. BALB/c mice were immunized with 9 HSP105 peptides. Using the ^51^Cr release assay, sensitized spleen cells that had been stimulated *in vitro* with each HSP105 peptide (10 μmol/l) and cultured for 5 days with 100 U/ml interleukin-2 were examined for CTL activity against C26 cells and C26 cells transfected with HSP105 siRNA (C26/HSP105 RNAi). Values represent the percentage of specific cell lysis, based on the mean values from triplicate assays.

There were similar structural motifs within the peptides that bound to human HLA-A24 and mice K^d^. We selected those peptides with binding motifs for both HLA-A24 and K^d^ molecules and prepared 9 different synthetic peptides (HSP105-1-9). When we tested these peptides for their potential to induce *in vitro* tumor reactive CTLs in spleen cells derived from BALB/c mice immunized with the HSP105 peptides, only the HSP105 24-1 peptide-induced CTLs showed specific cytotoxicity against C26 tumors (HSP105^+^, H-2K^d^) (Fig. 2). The cytotoxicity against C26 was attenuated by HSP105 siRNA. These findings indicate that the HSP105 A24-1 peptide has the capacity to induce tumor reactive CTLs and that peptide vaccination-primed CTLs are reactive to this peptide *in vivo*. We would expect this HSP105 A24-1 (NYGIYKQDL) peptide to also be an epitope for human CTLs.

### HSP105-reactive CTLs from PBMCs of HLA-A2-positive colorectal cancer patients and CTLs induce cytotoxicity against HSP105-expressing cancer cells

**Figure 3:**
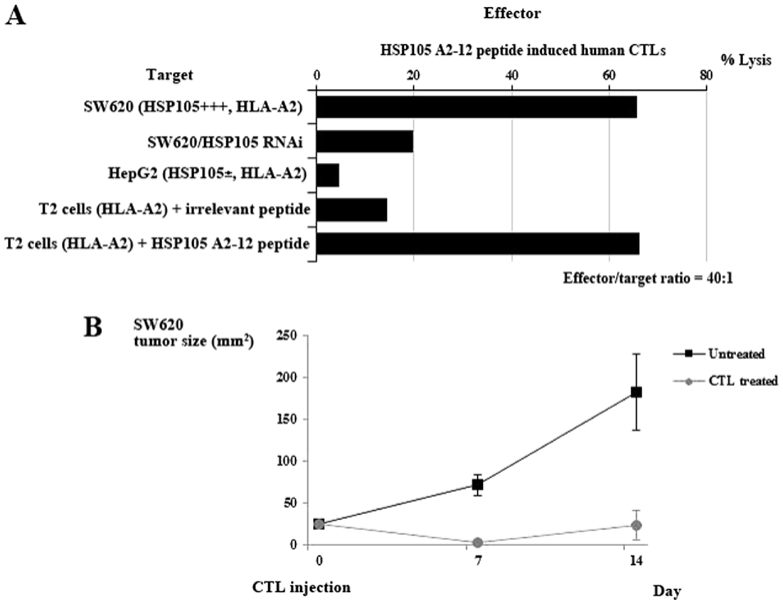
CTL induction from PBMCs of HLA-A2-positive cancer patients. (A) HSP105 peptide-reactive CTLs were generated from CD8^+^ T cells of HLA-A2^+^ colorectal cancer patients. After three or four stimulations with autologous monocyte-derived DCs pulsed with the HSP105 A2-12 peptides, the CTLs were subjected to a standard ^51^Cr release assay at the indicated effector/target ratio (40/1). Their cytotoxicity against SW620 cells (HSP105^+++^, HLA-A2), SW620 cells transfected with HSP105 siRNA (HSP105^−^), HepG2 cells (HSP105^±^, HLA-A2), T2 cells pulsed with an irrelevant peptide (HSP105^−^, HLA-A2) and T2 cells pulsed with the HSP105 A2-12 epitope peptide were all examined by ^51^Cr release assay. Values represent the percentage of specific cell lysis, based on the mean values from triplicate assays. (B) There was marked growth inhibition of SW620 cells (HSP105^+^) engrafted into nude mice after intratumoral injection of human CTLs induced by the HSP105 peptides. When tumor size reached 25 mm^2^ on day 9 after s.c. tumor implantation, human CTLs (3×10^6^) reactive to the HLA-A2-restricted HSP105 peptide, generated from an HLA-A2^+^ donor, were i.t. inoculated. Tumor sizes in nude mice administered the HSP105 epitope peptide-induced CTL lines (n=3), or no treatment (n=3), are shown. The mean tumor size (mm^2^) for each group of mice was expressed, and bars represent SD.

We generated a CTL line from PBMCs of colorectal patients by stimulation with the HSP105 A2-12 peptide. As shown by ^51^Cr release assays, the resulting CTLs showed HSP105-specific cytotoxicity against SW620 cells (HSP105^+++^, HLA-A2) and against T2 cells pulsed with the HSP105 A2-12 peptide (HSP105^−^, HLA-A2), but not against HepG2 cells (HSP105^±^, HLA-A2) or T2 cells pulsed with an irrelevant peptide (Fig. 3A). HSP105 siRNA decreased the cytotoxicity against SW620 cells. We investigated the effects of the HSP105 A2-12 peptide-reactive CTL lines on the mice implanted with the SW620 cells. Fourteen days after inoculation of HSP105 A2-12 peptide-reactive CTLs, there was an apparent reduction in tumor size in the SW620 compared to that in untreated mice (Fig. 3B). These results clearly indicate the efficacy of HSP105 A2-12 (KLMSSNSTDL) peptide-reactive CTL injection therapy for HSP105^+^ tumors in mice.

### Detection of HSP105-specific CTLs in peripheral blood of pre-surgical patients with colorectal cancer

Our results suggest that the four peptides, HSP105 A2-7 (RLMNDMTAV), HSP105 A2-12 (KLMSSNSTDL), HSP105 A24-1 (NYGIYK QDL) and HSP105 A24-7 (EYVYEFRDKL), are HSP105-derived, HLA-A2, or HLA-A24-restricted CTL epitopes. To determine the frequencies of the HSP105-derived T cells specific for these peptide in pre-surgical colorectal cancer patients, we analyzed the PBMC responses for each peptide using the ELISPOT assay. HSP105 expression was detected in 20 of 21 (95%) patients, consistent with previous studies (4). HSP105-specific T cells secreting IFN-γ were detected in patients stimulated with the HSP105 A2-7 (4 patients), HSP105 A2-12 (6 patients), HSP105 A24-1 (2 patients) and HSP105 A24-7 (6 patients) peptides (Table II). ELISPOT assay detected positive IFN-γ responses to at least one of the HSP105-derived peptides in PBMCs in 14 of the 21 patients. In contrast to the results for colorectal cancer patients, the 4 peptides were not recognized by PBMCs from healthy donors. Both the ratio of normal donors who showed positive T-cell responses to CMV-derived peptides and the frequencies of the specific T cells were identical to those of the colorectal cancer patients (data not shown).

### HSP105-derived peptide immunization does not induce autoimmunity in HLA-A2 Tgm

**Figure 4:**
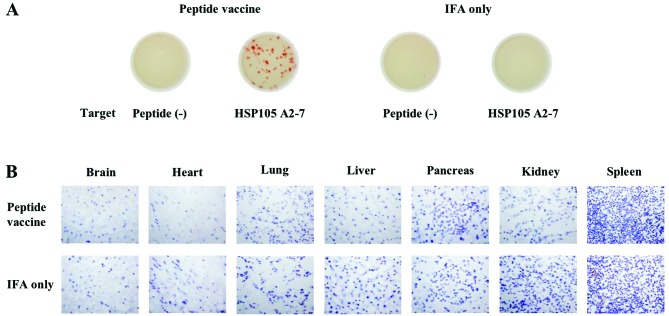
The HSP105 peptide vaccine induces HSP105 peptide-specific CTLs, while CD8 T cells do not infiltrate into normal tissues. (A) HSP105 peptide-specific CTLs were induced in the spleen cells of immunized mice with the HSP105 peptide vaccine. IFN-γ ELISPOT assays were performed using BM-DCs pulsed with HSP105 A2-7 and non-pulsed BM-DCs as target cells. The representative data are shown (n=6). (B) Immunohistochemical staining with anti-CD8 mAb was performed in tissue specimens of HLA-A2 Tgm immunized with the HSP105 A2-7 and A2-12 peptides. The tissue specimens were removed and analyzed 7 days after the second vaccination (original magnification, ×400). The representative data are shown (n=3).

HSP105 in normal adult mice is expressed in only certain tissues, and expression in these tissues is less than that in C26 tumor cells, suggesting a low risk of damage to normal tissues posed by HSP105 antigen-induced immune responses (6). To investigate whether immunization of the mice with HSP105-derived peptides causes autoimmunity, HLA-A2 Tgm were immunized with the HSP105 A2-7 and A2-12 peptides emulsified in incomplete Freund’s adjuvant at 7-day intervals and then sacrificed 7 days after the second vaccination. Using the IFN-γ ELISPOT assay, we confirmed the induction of HSP105 peptide-specific CTLs in the spleen cells of immunized mice (Fig. 4A). We did not detect any pathological changes, such as CD8^+^ lymphocyte infiltration or tissue destruction/repair, in the brain, heart, lung, liver, pancreas, or kidney of HLA-A2 Tgm (Fig. 4B). These results indicate that the HSP105 peptide-reactive CD8^+^ CTLs did not attack the healthy tissue specimens that we evaluated.

## Discussion

Heat shock proteins (HSPs) have essential functions in the regulation of protein folding, conformation, assembly and sorting. They function as molecular chaperones to maintain the native conformational states of proteins, preventing protein aggregation (18). HSPs are classified into several families based on their molecular weight, including HSP105/110, HSP90, HSP70, HSP60, HSP40 and HSP27 (19). HSP105 is a stress protein within the HSP105/110 family that we previously reported to be overexpressed in a variety of human cancers but with little to no expression in normal tissues, aside from the testis. Thus, HSP105 presents a promising candidate for a target antigen in cancer immunotherapy (3–7). In particular, HSP105 is specifically overexpressed in colorectal cancer (83%) (4). Furthermore, HSP105 is expressed in highly metastatic colon cancer cell lines and its expression is correlated with advanced clinical cancer stages and positive lymph node involvement (20). When considering immunogenic target molecules for cancer immunotherapy, it is important to select a tumor antigen that does not run the risk of becoming lost during immunoediting (21). We reported previously that siRNA-mediated suppression of HSP105 protein expression induced apoptosis in various types of cancer cells, but not in fibroblasts (12). Therefore, it is possible that tumor cells do not lose HSP105 expression, allowing for continued growth.

Advances in molecular biology and tumor immunology have paved the way for identification of a large number of tumor-associated antigens (TAAs) and antigenic peptides recognized by tumor reactive CTLs; hence, peptide-based cancer immunotherapy has become an intensely studied field (22,23). Several HSPs, including HSP70, HSP90 and gp96, bind and deliver (through receptor-mediated endocytosis of HSP) antigenic peptides to the antigen-processing pathway of antigen-presenting cells (APCs) and these peptides are then presented on major histocompatibility complex (MHC) class I molecules. This HSP-mediated pathway has been demonstrated to evoke potent antiviral and antitumor immune responses (24). On the other hand, many researchers have identified MHC class I-presenting peptide epitopes derived from HSP (25). Furthermore, HSP105 itself may induce CD8^+^ T cells to become reactive towards tumor cells that express HSP105, using HSP105-DNA and HSP105-pulsed DC vaccines in mice (6–8).

We found 4 peptides [HSP105 A2-7 (RLMNDMTAV), HSP105 A2-12 (KLMSSNSTDL), HSP105 A24-1 (NYGI YKQDL) and HSP105 A24-7 (EYVYEFRDKL)] to be potential HSP105-derived, HLA-A2 or A24-restricted CTL epitopes. There was a discrepancy between the expected HSP105 CTL epitopes in Tgm and in PBMCs of colorectal cancer patient. To identify the HSP105-derived CTL epitope peptides, we analyzed the PBMC responses to each of the 4 peptides in colorectal cancer patients using the *ex vivo* IFN-γ ELISPOT assay.

In this study, we used an *ex vivo* assay to detect HSP105-specific IFN-γ-secreting T cells in PBMCs from 14 of 21 pre-surgical patients with colorectal cancer. Generally, CTLs specific for tumor antigens cannot be detected directly *ex vivo*; rather only after expansion by repeated *in vitro* stimulation with the antigenic peptide in the appropriate antigen-presenting cells. This is attributed to assay sensitivity and the low frequency of tumor antigen-specific CTLs (26). HSP105-specific CTLs in PBMCs, which can be detected directly *ex vivo* without *in vitro* stimulation, provide strong immunological evidence of HSP105-derived CTL epitopes, which we were able to identify in this study. However, because the prognosis of the pre-surgical patients was affected by various factors, it was difficult to evaluate the correlation between a positive CTL response before surgery and clinical improvement at the present stage; an increase in the number of patients at each stage and further analyses of this relationship are necessary.

Although the SEREX method facilitated the identification of tumor antigens that could be recognized by antibodies and CD4^+^ T cells, few of their T-cell epitopes have been determined (27). We previously reported in mice that HSP105-DNA and HSP105-pulsed DC vaccines induced a reaction in CD4^+^ T cells and CD8^+^ T cells towards tumor cells expressing HSP105 (6–8). HSP105 was identified by SEREX (3) and thus, HSP105-specific CD4^+^ T cell reactions may be induced by HSP105 immunization. It was shown that antigen-specific CD4^+^ T cells are required to activate memory CD8^+^ T cells into fully functional effector killer cells (28). We are now preparing a clinical trial to investigate HSP105-based immunotherapy for HSP105-expressing tumors, including those from colorectal cancer. We plan to use the HSP105 epitope peptides identified in this study as an initial attempt. We expect that HSP105-based immunotherapy will be a novel treatment strategy for colorectal cancer patients.

## Figures and Tables

**Table I:** HSP105-derived peptides conserved between human and mouse HSP105 predicted to bind to HLA-A2 or HLA-A24.

Peptides	Position	Subsequent residue listing	HLA-A2 binding score
HSP105 A2-4	120–128	MLLTKLKET	107
HSP105 A2-5	141–149	VISVPSFFT	55
HSP105 A2-6	155–163	SVLDAAQIV	37
HSP105 A2-7	169–177	RLMNDMTAV	591
HSP105 A2-9	202–210	DMGHSAFQV	21
HSP105 A2-10	222–230	VLGTAFDPFL	759
HSP105 A2-12	275–284	KLMSSNSTDL	276
HSP105 A2-13	276–284	LMSSNSTDL	26
HSP105 A2-14	300–309	KMNRSQFEEL	50
HSP105 A2-15	304–313	SQFEELCAEL	32
HSP105 A2-16	313–321	LLQKIEVPL	36
HSP105 A2-19	434–442	FLRRGPFEL	43
HSP105 A2-20	458–467	KIGRFVVQNT	76
HSP105 A2-25	668–676	LLTETEDWL	401
HSP105 A2-26	675–684	WLYEEGEDQA	146
HSP105 A2-29	757–765	EVMEWMNNV	15

Peptides	Position	Subsequent residue listing	HLA-A24 binding score

HSP105 A24-1	180–188	NYGIYKQDL	240
HSP105 A24-2	214–223	AFNKGKLKVL	30
HSP105 A24-3	251–260	KYKLDAKSKI	110
HSP105 A24-4	305–313	QFEELCAEL	47
HSP105 A24-5	433–442	TFLRRGPFEL	33
HSP105 A24-6	613–622	MYIETEGKMI	90
HSP105 A24-7	640–649	EYVYEFRDKL	330
HSP105 A24-8	725–733	HYAKIAADF	140
HSP105 A24-9	739–748	KYNHIDESEM	82

The binding scores were estimated by using BIMAS software: http//bimas.dcrt.nih.gov/cgi-bin/molbio/ken_parker_comboform.

**Table II:** Expression of HSP105 in colorectal cancer tissue and quantification of HSP-specific CTLs in colorectal cancer patients.

						cSpot number of peptide-specific CTLs				
						
HLA-A2-positive patients	Age (yrs.)	Gender	HLA	Stage^a^ of tumor	HSP105 expression^b^	HSP105 A2-7		HSP105 A2-12		CMV
1	62	M	0201/2601	IIIB	++	27	+	126	+	160
5	79	M	0207/1101	IIIB	++	0	−	2	−	10
6	51	M	0201/0206	I	+	0	−	49	+	136
8	55	M	0206/2402	I	±	0	−	0	−	66
11	69	M	0206/2402	IIIC	+	143	+	0	−	0
12	61	M	0201/3303	I	±	2	−	45	+	367
13	64	F	0201/2601	IIIC	±	0	−	2	−	254
14	66	M	0206/2402	IIIC	−	13	+	0	−	58
15	78	M	0201/1101	IIA	+	0	−	5	+	57
16	51	F	0206/2601	IV	±	31	+	7	+	15
17	63	F	0206/1101	IIA	++	0	−	25	+	96

HLA-A2402-positive patients						HSP105 A24-1		HSP105 A24-7		CMV

2	64	F	2402	IV	++	2	−	44	+	6
3	60	M	2402/3101	IIIC	++	0	−	0	−	11
4	71	F	2402/3101	IIA	++	25	+	51	+	12
7	47	M	2402/3101	IIIA	++	4	−	6	+	3
9	66	M	2402	IV	++	8	+	6	+	7
10	60	M	2402/3101	I	++	1	−	19	+	26
18	64	M	1101/2402	IV	+	0	−	2	−	40
20	46	F	1101/2402	IIIB	++	4	−	7	+	5
21	66	F	2402	I	++	3	−	0	−	38

F, female; M, male.

a Stage, staging was performed according to the TNM classification (Union for International Cancer Control; UICC).

b HSP105 expression, staining intensity of tumor cells was scored on a scale according to the following four grades: −, absent; ±, weak; +, moderate; ++, strong.

c Spot number indicates the number of peptide-specific CTLs calculated by subtracting the spot number in a well of no peptide. −, Spot number <5; +, Spot number ≥5.
